# Pancreatitis and Myocardial Infarction as Complications of Thrombotic Thrombocytopenic Purpura: A Case Report

**DOI:** 10.1002/ccr3.73492

**Published:** 2026-09-11

**Authors:** Farid Poursadegh, Motahhareh Karimoddini, Mohsen Seddigh‐shamsi, Najme Mohajer

**Affiliations:** ^1^ Lung Disease Research Center Mashhad University of Medical Sciences Mashhad Iran; ^2^ Internal Medicine Department, Faculty of Medicine Mashhad University of Medical Sciences Mashhad Iran; ^3^ Department of Hematology‐ Oncology, Faculty of Medicine Mashhad University of Medical Sciences Mashhad Iran; ^4^ Resident of Internal Medicine Mashhad University of Medical Sciences Mashhad Iran

**Keywords:** acute pancreatitis (AP), ADAMTS13, myocardial infarction (MI), plasma exchange, thrombocytopenia, thrombotic thrombocytopenic purpura (TTP)

## Abstract

Thrombotic thrombocytopenic purpura (TTP) is a rare disease with a mortality rate of 90% if not treated promptly. Due to limited clinical experience and sometimes atypical presentation, early detection of TTP is not always easy. The pathophysiological mechanisms underlying TTP can accelerate thrombus formation and vascular occlusion, potentially leading to ischemic damage in various organs, including the heart and pancreas. Considering that the complications caused by these disorders can be life‐threatening, the simultaneous manifestation of acute pancreatitis (AP), TTP, and heart damage in the patient may cause a significant risk of mortality if not treated in time and adequately. We report a case involving a 43‐year‐old patient who presented with symptoms of myocardial infarction (MI) and acute pancreatitis (AP) secondary to thrombotic thrombocytopenic purpura (TTP). The patient was diagnosed based on clinical manifestations, laboratory findings, and imaging studies. Immediate intervention included plasma exchange and supportive care, which led to the stabilization of the patient's condition. This case highlights the importance of actively assessing and monitoring for organ involvement, including pancreatic and cardiac complications, in patients with TTP. Early diagnosis and prompt treatment are critical in reducing the risk of mortality associated with these severe complications.

## Key Clinical Message

Thrombotic thrombocytopenic purpura (TTP) can present with serious complications like pancreatitis and myocardial infarction. Early recognition and treatment with plasma exchange are critical to improving patient outcomes.

## 1 Introduction

Thrombotic Thrombocytopenic Purpura (TTP) is a potentially life‐threatening condition characterized by severe thrombocytopenia and microangiopathic hemolytic anemia. The primary pathophysiological mechanism is reduced ADAMTS13 activity, which can be due to inherited gene mutations (hereditary TTP) or the formation of inhibitory autoantibodies (acquired TTP) [1]. ADAMTS13 is a von Willebrand factor (VWF)‐cleaving protease that prevents the accumulation of ultra‐large VWF molecules by cleaving them into smaller fragments. In the absence of ADAMTS13, ultra‐large VWF molecules accumulate within vessels, forming platelet‐rich microthrombi. Consequently, diminished blood supply to tissues can result in multi‐organ damage [2, 3]. Given the systemic nature of TTP, it can lead to complications in various organs. Neurologic and cardiac involvement are among the most clinically significant manifestations and represent major causes of morbidity and mortality in TTP [1]. Acute pancreatitis (AP) is an uncommon complication of TTP, whereas cardiac involvement, although variably reported, is increasingly recognized in clinical series. Understanding the broader context of AP and MI helps underscore their clinical relevance in TTP patients. The annual incidence rate of AP ranges between 4.9 and 35 per 100,000 individuals, with mortality rates of 1.5% and 30% in mild and severe cases, respectively [4, 5, 6]. Hence, early diagnosis and intervention are vital in managing these patients. AP can be attributed to several etiologies, including obstruction of the pancreatic duct due to gallstones (38%), alcohol consumption (36%), and hypertriglyceridemia (up to 4%) [7]. In rare instances, pancreatitis can develop secondary to Thrombotic Thrombocytopenic Purpura/Hemolytic Uremic Syndrome (TTP/HUS). Approximately 2% of patients with TTP/HUS develop acute pancreatitis [8, 9]. This phenomenon is hypothesized to occur due to the necrosis of pancreatic cells resulting from occlusion of pancreatic arterioles by platelet‐rich thrombi, although the exact mechanism remains unclear [10]. Cardiac involvement in TTP is well documented and may range from asymptomatic troponin elevation to overt myocardial infarction due to microvascular thrombosis. Reported frequencies of cardiovascular complications vary widely (9.5%–77%), largely depending on definitions and diagnostic criteria used in different studies [11]. However, myocardial infarction as the initial presenting feature of TTP remains uncommon. We present a rare case of a 43‐year‐old man with acute pancreatitis and myocardial infarction (MI) as the initial presentation of TTP.

### 1.1 Case History and Physical Examinations

A 43‐year‐old man with no medical history went to the Emergency Department 2 days before being admitted to the hospital with the main complaint of severe abdominal pain in the epigastric area with radiation to the back, nausea, vomiting, cold sweat, and headaches. The patient had no history of alcohol or drug use. On examination, the patient was conscious and his vital signs were stable. The conjunctiva was pale, and the sclera showed evidence of icterus. Tenderness was observed in the epigastrium. Other examinations had normal results.

### 1.2 Investigations, Diagnosis and Treatment

**FIGURE 1:**
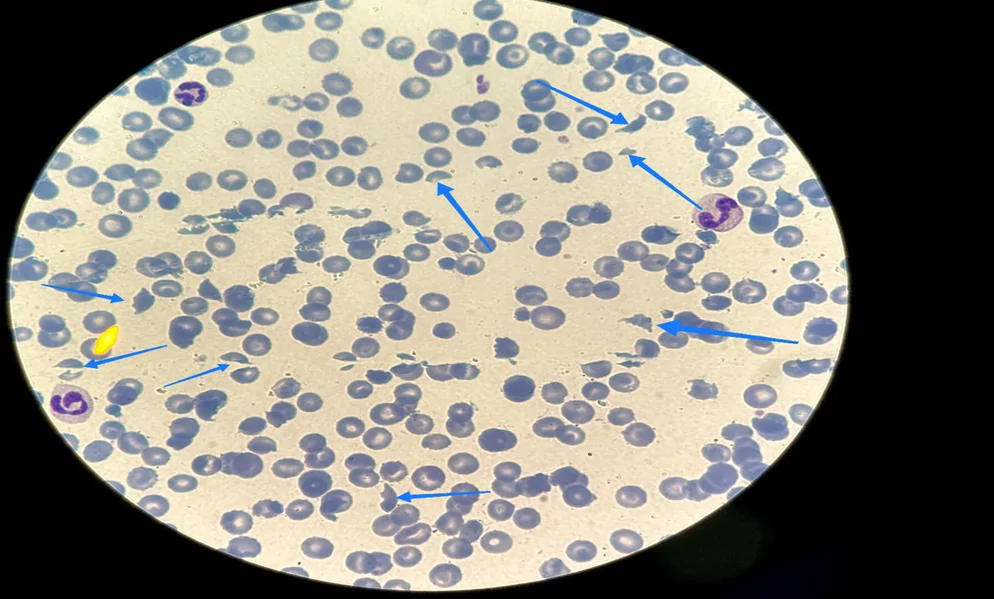
The Schistocytes (blue arrows) were found in peripheral blood smear (H&E staining with 100× magnification).

**FIGURE 2:**
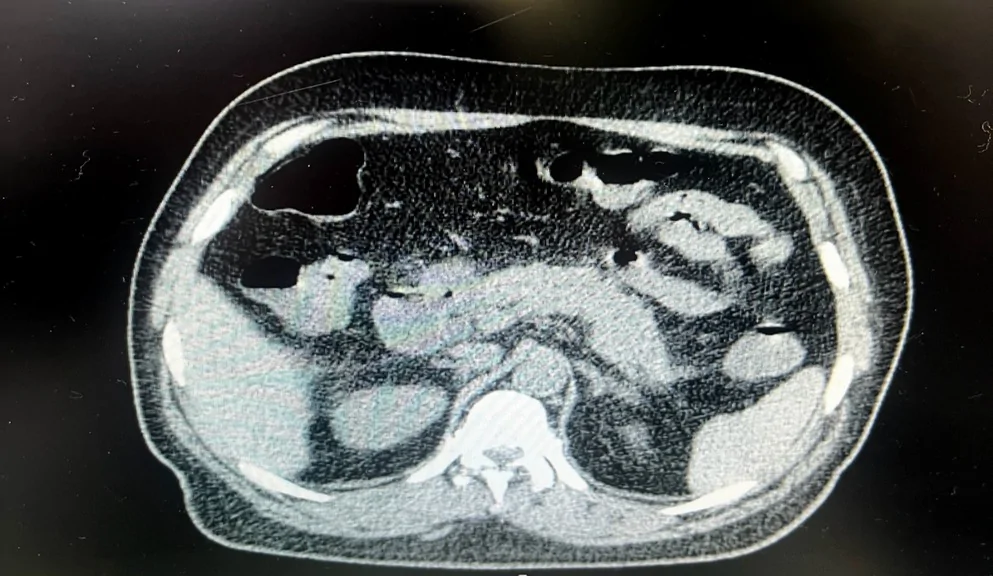
Abdomen Computed Tomography (CT) Scan; Pancreatic swelling and surrounding fat stranding.

Laboratory tests revealed the following results: hemoglobin: 6.7 g/dL; MCV: 88 fL; platelets: 13,000/L; total bilirubin: 5.3 mg/dL with indirect bilirubin: 3.2 mg/dL; LDH: 3981 U/L; retic percentage: 3%; International Normalized Ratio (INR): 1.3; creatinine: 1.3 mg/dL; cardiac troponin I level: 410 ng/mL; amylase: 390 U/L; and lipase: 510 U/L. Direct and indirect Coombs tests were negative. The patient's peripheral blood smear showed the presence of schistocytes (Figure 1). ADAMTS‐13 activity level was 0% with elevated inhibitor titer (1.02 BU/Ml: Normal range: < 0.5 BU/mL) and negative antinuclear antibody level. The patient's electrocardiogram showed normal sinus rhythm without ST segment changes, and echocardiography showed normal findings. A diagnosis of TTP was made, and plasma exchange was rapidly initiated based on a PLASMIC score of 7. Methylprednisolone 1000 mg per day intravenously was prescribed for 3 days. Due to increased serum amylase and lipase levels, a computed tomography (CT) scan of the abdomen was performed, which showed a swollen pancreas with fatty fibers around it (Figure 2). An abdominal ultrasound showed the absence of radiolucent stones. Subsequent laboratory tests ruled out hypercalcemia and hypertriglyceridemia as potential causes of pancreatitis. A CT scan of the brain was normal. Based on the patient's symptoms, cardiac troponin I level was measured again, which was 1100 ng/mL. A consultation with a cardiologist revealed that a non‐ST‐segment elevation myocardial infarction had occurred. After three plasma exchanges, the patient's platelet count reached 52,000/L, and Tab Aspirin (ASA) and heparin were started at a therapeutic dose. After the seventh session of plasma exchange, the patient's platelets reached more than 150,000/uL, and after the eighth session, it was 190,000/uL. At the time of discharge, it was 205,000/uL. The patient's LDH also decreased to 446 U/L. Finally, after eight sessions of plasma exchange and 3 days of methylprednisolone and serum therapy, all the patient's symptoms were resolved, and the patient was discharged with Clopidogrel, ASA, Atorvastatin, and oral Prednisolone 50 mg. The dose of prednisolone was gradually reduced over 4 weeks.

### 1.3 Outcome and Follow‐Up

During follow‐up periods at 1 and 6 months, the patient showed no signs of disease recurrence. The normalization and sustained maintenance of platelet count ≥ 150,000/uL for at least 48 h together with resolution of hemolysis fulfilled the criteria for clinical response, and the absence of recurrence after discontinuation of plasma exchange was consistent with clinical remission, according to the international consensus definitions for immune thrombotic thrombocytopenic purpura [12]. During hospitalization, serial electrocardiograms revealed no dynamic ischemic changes. Transthoracic echocardiography demonstrated preserved left ventricular systolic function without regional wall motion abnormalities. With appropriate management, cardiac troponin I levels progressively declined and reached 0.15 ng/mL at the time of discharge. At follow‐up evaluation, left ventricular function remained normal, and the patient exhibited no clinical signs or symptoms of heart failure or recurrent ischemia.

## 2 Discussion

The clinical spectrum of TTP‐related organ damage is broad, with microvascular thrombosis leading to diverse manifestations such as myocardial infarction and acute pancreatitis [13]. While AP is reported in only 1.7%–2.0% of cases, the underlying mechanism involves hyaline microthrombi in pancreatic small vessels causing ischemia and subsequent inflammatory changes [14]. In 2024, Wang et al. reported a 44‐year‐old man diagnosed with acute pancreatitis as a rare manifestation of TTP, who presented with initial symptoms of abdominal pain [15]. Harvey Olsen reported a case involving a 49‐year‐old male with TTP‐induced acute pancreatitis (AP), who exhibited symptoms of abdominal pain and bleeding gums. Histological evaluation of the patient's pancreatic parenchyma revealed focal hemorrhages, fat necrosis, and round cell infiltration. Additionally, the presence of the pancreatic enzyme elastase was noted in the pancreatic tissue, aligning with the characteristics of AP [10]. A separate review by Antes EH documented histological alterations in 63 TTP patients with confirmed AP. Of the nine individuals diagnosed with AP, five exhibited necrosis and two displayed hemorrhages in the pancreatic tissue, corroborating the aforementioned mechanism. Furthermore, an animal study demonstrated that thrombosis in pancreatic veins induced acute necrotizing hemorrhagic pancreatitis in dogs [16]. Swisher et al. detailed the cases of five patients who initially presented with acute pancreatitis and later exhibited signs of TTP (including thrombocytopenia, anemia, elevated LDH, and the presence of schistocytes in blood smears) within a span of 1–13 days [17].

MI due to TTP is extremely rare as the presenting event of TTP. In 2023, Mohamed et al. reported a 45‐year‐old woman who presented with symptoms of fever, myalgia, diffuse arthralgia, and decreased urination 3 days after the diagnosis of Non‐ST‐Segment Elevation Myocardial Infarction and undergoing percutaneous coronary intervention (PCI). Attention to laboratory tests and a low ADAMTS13 level with a high inhibitory titer led to the diagnosis of TTP [11]. Also, in 2022, Gheith et al. reported a 68‐year‐old woman who presented with an initial complaint of chest pain along with mental changes and fatigue, and a platelet count of 30,000/uL, with a non‐ST elevation myocardial infarction as an unusual presentation of TTP. She was treated with plasmapheresis and steroids [18]. Șalaru et al. [19], Ghodsi et al. [20], Takimoto et al. [21], and Dahal et al. [22] also reported myocardial infarction as a rare and early manifestation of TTP. In this report, at the same time as the diagnosis of TTP and AP for the patient, Non‐ST‐Segment Elevation Myocardial Infarction was also discussed. Cardiac complications caused by TTP have a high mortality rate, and early diagnosis and management of this disease are very important [11]. Studies have shown that TTP patients with high levels of positive cardiac biomarkers are at higher risk for severe complications and mortality. Troponin levels greater than 0.25 ng/mL are associated with a threefold increased risk of mortality in TTP patients [23]. In this report, the troponin level was first 410 ng/mL and then 1100 ng/mL. Management of myocardial infarction in TTP patients can be challenging due to low platelet counts. Standard recommendations suggest a cardiac workup with clinical examination, Electrocardiogram (EKG), echocardiography, and serum evaluation of cardiac enzymes. In TTP patients with myocardial damage, immediate plasmapheresis is necessary to prevent further cardiac damage and mortality. Additionally, due to the increased risk of fatal arrhythmia, continuous heart monitoring is necessary for these patients [11]. One limitation of our report is that ADAMTS13 activity was not measured at follow‐up; therefore, we were unable to confirm test‐based remission, defined as normalization of ADAMTS13 activity. Clinical remission in our patient was defined as normalization of platelet count, absence of schistocytes in peripheral blood smear, and resolution of symptoms. Here we report a 43‐year‐old man with acute pancreatitis and myocardial infarction (MI) as potential manifestations of TTP‐related microvascular injury. This case report describes the occurrence of both acute pancreatitis and myocardial infarction as complications of TTP, emphasizing the critical importance of early diagnosis and intervention in the management of this disorder. It also provides insight into the challenges of diagnosing and treating TTP with unusual presentations such as pancreatitis and myocardial infarction. The complexity of managing TTP, especially when it is associated with multiorgan involvement, underscores the need for physicians to be vigilant in caring for such complications.

## Author Contributions

**Farid Poursadegh:** conceptualization, project administration, supervision, validation, visualization. **Motahhareh Karimoddini:** conceptualization, data curation, investigation, methodology, resources, software, writing – original draft, writing – review and editing. **Mohsen Seddigh‐shamsi:** investigation, methodology, project administration, resources, validation, visualization. **Najme Mohajer:** data curation, investigation, project administration, visualization.

## Funding

The authors have nothing to report.

## Consent

Written informed consent was obtained from the patient to publish this report in accordance with the journal's patient consent policy.

## Conflicts of Interest

The authors declare no conflicts of interest.

## Data Availability

The data that support the findings of this study are available from the corresponding author upon reasonable request.
